# Cardiovascular, Metabolic Effects and Dietary Composition of Ad-Libitum Paleolithic *vs.* Australian Guide to Healthy Eating Diets: A 4-Week Randomised Trial

**DOI:** 10.3390/nu8050314

**Published:** 2016-05-23

**Authors:** Angela Genoni, Philippa Lyons-Wall, Johnny Lo, Amanda Devine

**Affiliations:** 1 School of Medical and Health Sciences, Edith Cowan University, Perth 6027, Australia; p.lyons-wall@ecu.edu.au (P.L.-W.); a.devine@ecu.edu.au (A.D.); 2 School of Science, Edith Cowan University, Perth 6027, Australia; j.lo@ecu.edu.au

**Keywords:** Paleolithic, AGHE, cardiovascular, obesity, metabolic

## Abstract

(1) Background: The Paleolithic diet is popular in Australia, however, limited literature surrounds the dietary pattern. Our primary aim was to compare the Paleolithic diet with the Australian Guide to Healthy Eating (AGHE) in terms of anthropometric, metabolic and cardiovascular risk factors, with a secondary aim to examine the macro and micronutrient composition of both dietary patterns; (2) Methods: 39 healthy women (mean ± SD age 47 ± 13 years, BMI 27 ± 4 kg/m^2^) were randomised to either the Paleolithic (*n* = 22) or AGHE diet (*n* = 17) for four weeks. Three-day weighed food records, body composition and biochemistry data were collected pre and post intervention; (3) Results: Significantly greater weight loss occurred in the Paleolithic group (−1.99 kg, 95% CI −2.9, −1.0), *p* < 0.001). There were no differences in cardiovascular and metabolic markers between groups. The Paleolithic group had lower intakes of carbohydrate (−14.63% of energy (E), 95% CI −19.5, −9.7), sodium (−1055 mg/day, 95% CI −1593, −518), calcium (−292 mg/day 95% CI −486.0, −99.0) and iodine (−47.9 μg/day, 95% CI −79.2, −16.5) and higher intakes of fat (9.39% of E, 95% CI 3.7, 15.1) and β-carotene (6777 μg/day 95% CI 2144, 11410) (all *p* < 0.01); (4) Conclusions: The Paleolithic diet induced greater changes in body composition over the short-term intervention, however, larger studies are recommended to assess the impact of the Paleolithic *vs.* AGHE diets on metabolic and cardiovascular risk factors in healthy populations.

## 1. Introduction

The theory behind the Paleolithic diet, that the introduction of agriculture around 10,000 years ago is at odds with human genetic makeup and leads to the development of obesity and chronic diseases [1], has received growing media coverage and celebrity endorsements in Australia. As described by Cordain [2], the diet encourages consumption of fruits, vegetables, lean meats, nuts and eggs. All products of agricultural origin, grains, legumes and dairy are excluded. The Australian National Health and Medical Research Council (NHMRC) recently released the revised Australian Dietary Guidelines (ADG) and Australian Guide to Healthy Eating (AGHE) [3]. The ADG aim to promote health and wellbeing while reducing the risk of diet-related diseases [3]. The exclusion of two food groups (grains and dairy) on the Paleolithic diet contradicts evidence based recommendations from the NHMRC [3] and therefore requires investigation regarding the health effects, nutritional adequacy and impacts on disease risk factors. There is limited literature on the efficacy of the Paleolithic diet in healthy subjects, however, short duration, uncontrolled studies [4,5,6] show promising results on cardiovascular and metabolic risk factors. The aim of the study was to compare the efficacy of ad-libitum Paleolithic and AGHE diets on anthropometric, cardiovascular and metabolic risk factors in healthy women aged 18–70 years, and to assess energy and nutrient intake.

## 2. Materials and Methods

### 2.1. Study Population

**Figure 1:**
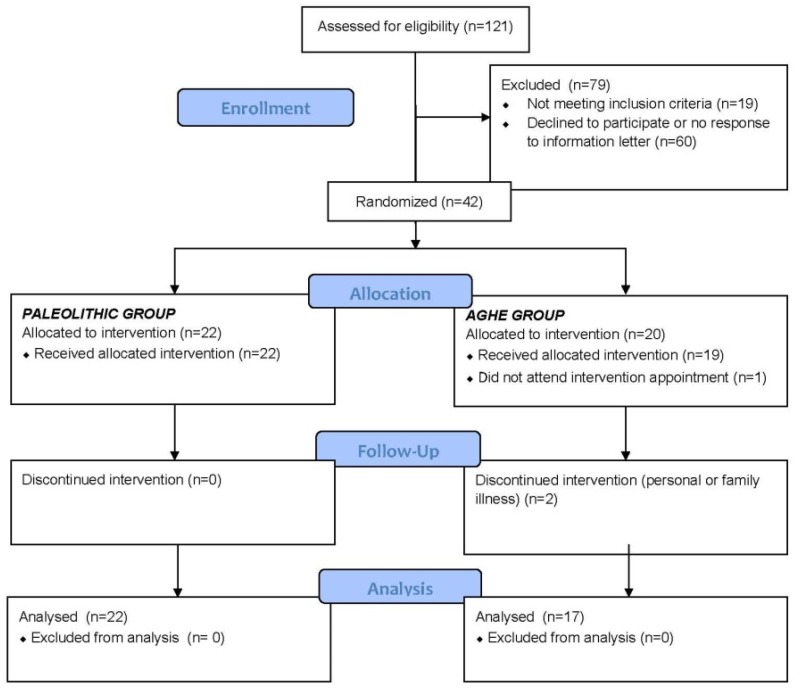
Flow diagram of subjects’ participation in the trial.

Forty-two women provided consent and were recruited for the study from September 2013 to May 2014. One participant did not attend the baseline appointment and two withdrew for illness or personal reasons, during the intervention period (AGHE group) (Figure 1). The remaining 39 women completed the study and were included in the analyses.

Prior to recruitment, an *a priori* power analysis was performed [7]. As this study was not advertised as a weight loss program, power calculations were based on reductions to total cholesterol as reported previously in healthy subjects consuming a Paleolithic diet [4,6]. Sample size was estimated using 5% α-error and 80% power between groups, based on reductions to total cholesterol. This provided a sample requirement of 17 subjects per group. Allowing for a 20% non-compliance rate, we aimed to recruit 21 subjects per group. *Post hoc* effect sizes were calculated for outcome variables that showed significant changes. Inclusion criteria included: willingness to change dietary patterns; non-smoker and non-participation in any other studies. Exclusion criteria were: current low carbohydrate or Paleolithic diet; medication for hypertension, hyperglycaemia or hyperlipidaemia; gastrointestinal disease; renal or liver disease; cardiovascular disease; coeliac disease or allergies to eggs, fish, dairy, nuts or wheat.

The study had ethics approval from the Edith Cowan University Human Research and Ethics Committee (Project 10176) and was registered on the Australian and New Zealand Register of Clinical Trials (ACTRN12615000246583).

### 2.2. Anthropometric and Biochemical Assessment

Pre and post intervention, waist circumference, stature and weight were measured by the same assessor, trained in anthropometry, to the nearest 0.1 cm and 0.1 kg respectively [8] with a measuring tape, SECA 763 digital column scale and stadiometer (SECA Ltd., Chino, CA, USA). Subjects voided prior to measurements, were fasted following blood testing, wore light clothing, no shoes and attended the clinic at the same time of day for both pre and post measures. Fat mass percentage was estimated in rested supine subjects using a single frequency bioelectrical impedance (BIA) analyser (Impedimed Imp DF50, Impedimed Ltd., Pinkemba, Australia). Following a 5-min supine rest period, systolic and diastolic blood pressures were recorded in duplicate 1-min apart, on the right arm using an Omron IA1B Automated Blood Pressure Monitor (Omron Healthcare Ltd., Kyoto, Japan).

Fasting blood and 24-h urine samples were collected at baseline and day 28 and analysed using standard enzymatic techniques with the Abbott Architect c16000 assay (Abbott Laboratories, IL, USA). Samples were analysed by Pathwest, an accredited National Association of Testing Laboratories (NATA) laboratory (Nedlands, Western Australia), for total cholesterol (coefficient of variation (CV_a_) 1.32% and 0.95% at concentrations of 2.95 and 6.6 mmol/L respectively), calculated low density lipoprotein (LDL) cholesterol, high density lipoprotein (HDL) cholesterol (CV_a_ 2.79% and 1.79% at 0.86 and 1.95 mmol/L), triglycerides (TG) (CV_a_ 2.04% and 1.54% at 1 and 2.02 mmol/L), glucose (CV_a_ 2.03% and 1.28% at 4.84 and 16.6 mmol/L), urinary sodium (CV_a_ 2.6% and 0.84% at 77 and 168 mmol/L) and creatinine (CV_a_ 2.6% and 1.9% at 5.2 and 11.2 mmol/L), insulin (CV_a_ 4.50% and 3.45% at 14.1 and 133 mU/L) and red cell folate (CV_a_ 17.37% and 6.16% at 55 and 400 nmol/L) were analysed using the Abbott Architect i2000SR assay (Abbott Laboratories, Abbott Park, IL, USA); serum α, β carotene and lycopene were analysed using National Institute of Science and Technology Standard 968e methods [9].

### 2.3. Dietary Intervention

Forty-six identical envelopes containing 23 of each of the dietary patterns were sealed and mixed thoroughly prior to subject recruitment. Subjects were randomised immediately prior to their baseline measures in a non-blinded study design, which included a dietary consultation. Those in the Paleolithic group were provided with meal ideas obtained from “The Paleo Diet” book [2] and advised to consume lean meats, fish, eggs, nuts, fruits and vegetables, and small amounts of olive or coconut oils. Grains, cereals and dairy products were not permitted. Dairy products were replaced with unsweetened almond milk. Sugarless black coffee and tea were allowed. All vegetables were permitted on the diet, except for corn, white potatoes and legumes. To ensure adequate carbohydrate, additional fruit was recommended. Dried fruit was limited to one tablespoon per day. The AGHE group were counselled on how to achieve food variety in line with the five food groups and Australian dietary recommendations; increasing vegetable and fruit intake and whole grain products, reducing fat intake, and consuming low fat dairy products. Advice was also given to reduce intake of discretionary food items such as cakes, biscuits, sugary drinks and candy. Subjects received the AGHE plate poster and fridge magnet [3]. Both groups consumed *ad libitum* diets and were contacted weekly for support and/or advice regarding food choices.

### 2.4. Dietary Assessment

Dietary intake was assessed with a 3-day weighed food record immediately prior to commencing the intervention and on days 26–28. Participants recorded all food and drink consumed for three consecutive days (two week days and one weekend day). Participants were instructed not to alter what they ate or drank during this time and to record using the electronic food scales (accurate to 1 g) provided or household measures (metric cups and spoons). Food records were reviewed with participants to clarify types and amount of food or beverages recorded. Analysis was undertaken by a nutritionist with competency in dietary assessment. Macro and micronutrient intakes were assessed using the AUSNUT2007 database (Foodworks 7 Professional, Xyris Software Ltd., Spring Hill, Australia).

#### Compliance

Study dietary compliance was assessed using a daily checklist of acceptable foods for each dietary pattern. Suitable foods consumed daily that aligned with the assigned dietary plan were checked on the list when eaten and additional items were recorded with a description and amount. All checklists were returned and evaluated for completeness. To determine compliance, items of additional foods that did not align with the assigned dietary pattern were coded with a serving amount. Five subjects consumed 1.5–2.5 serves per day of food outside the respective dietary patterns (Paleolithic *n* = 2, AGHE *n* = 3), however, despite the discretionary intake, the subjects had made changes to their dietary pattern and were therefore included in our data analysis. Excluding data from these subjects did not influence the significance of results.

### 2.5. Statistical Analysis

Dietary parameters, biochemistry and anthropometric data were analysed using SPSS v22 [10]. Normality was assessed using the Shapiro-Wilk test. Independent *t*-tests were initially conducted to assess differences between groups at baseline. Paired *t*-tests were conducted to compare changes within groups. Independent *t*-tests and Mann-Whitney U tests (for non-normally distributed data) were used to test for changes between groups. Univariate analysis of covariance (ANCOVA) was conducted to assess changes in weight, body composition and cholesterol levels whilst adjusting for weight loss, energy and nutrient intake. As multiple outcomes were analysed, the significance level was set at *p* < 0.01. Results with 0.01 < *p* < 0.05 were noted, but not reported as significant. Effect sizes (ES) were presented where appropriate. For the *t*-tests, small (S), medium (M) and large (L) Cohen’s effect sizes d were given by 0.3, 0.5 and 0.8 respectively [11]. For the Mann-Whitney U test, these levels were defined at 0.1, 0.3 and 0.5 [11].

## 3. Results

At baseline, the women were aged 47 ± 13 years, with BMI 27 ± 4 kg/m^2^, with no significant differences in subject or dietary characteristics, except for diastolic blood pressure, which was lower in the AGHE group (Paleolithic 77.8 ± 9 mmHg, AGHE 71.7 ± 7 mmHg, *p* = 0.03). Consumption of items outside the respective dietary regimens averaged 0.57 servings/day in the Paleolithic group and 1 serving/day in the AGHE group.

### 3.1. Anthropometric

Mean changes in anthropometric measurements are shown in Table 1. Body weight and waist circumference decreased in both groups, with a significantly greater decrease in the Paleolithic group compared to the AGHE group (*p* < 0.01, *d* = −0.82). The percentage weight loss (*p* < 0.01, *d* = −1.47), and reduction in fat mass (*p* < 0.01, *d* = −1.12) was greater in the Paleolithic group compared with the AGHE group. Loss of weight was in part associated with the percentage of energy derived from protein (*p* = 0.04), such that increased protein intake resulted in a trend towards greater weight loss. Percentage fat loss was greater, but not significant, in the Paleolithic group (*p* = 0.02), and not associated with reduction in energy intake.

### 3.2. Biochemicalu

Mean changes in biochemical measurements are shown in Table 2. There was a trend toward a greater change to red cell folate in the Paleolithic group (*p* < 0.05, *d* = 0.71) and serum β-carotene (*p* < 0.05, *d* = 0.67), when compared to the AGHE. Participants in both dietary groups experienced within group reductions to total cholesterol (*p* < 0.01, *d* = −0.74) and LDL cholesterol (Paleolithic *p* < 0.01, *d* = −0.91; AGHE trend 0.01 < *p* < 0.05, *d* = −0.53), however, there were no significant differences in these changes between the dietary groups. Further analysis showed the reduction in total cholesterol within groups was not associated with weight loss (*p* = 0.39). There were no significant differences between dietary groups in biomarkers of metabolism (fasting C-reactive protein, glucose, insulin, urinary sodium or creatinine) and no notable changes in serum α-carotene or lycopene.

### 3.3. Dietary Intake

#### 3.3.1. Macronutrients

Mean pre and post intake of macronutrients for the Paleolithic and AGHE groups are shown in Table 3. Between groups, there were significant differences in mean changes for percentages of daily energy derived from carbohydrate (*p* < 0.01, *d* = −1.96) and fat (*p* < 0.01, *d* = 1.08); differences in percentage of daily energy from protein (*p* < 0.05, *d* = 0.79) were not significantly different. Within the Paleolithic group, mean total energy intake was significantly lower post intervention (*p* < 0.01, *d* = −0.94) with significant increases in percentage of daily energy from protein (*p* < 0.01, *d* = 0.98), and fat (*p* < 0.01, *d* = 0.66). There was a significant decrease in percentage of daily energy from carbohydrate (*p* < 0.01, *d* = −1.36) and saturated fat (*p* < 0.01, *d* = −0.83). Within the AGHE group, there was a trend toward an increase in carbohydrate as percentage of daily energy (*p* < 0.05, *d* = 0.54) and a reduction in saturated fat intake (*p* < 0.05, *d* = −0.59). Dietary fibre intake was maintained in the Paleolithic group with no change in absolute daily intake. To account for the reduction in energy intake in both groups, fibre density was calculated (grams of fibre/energy in kJ × 100) and was significantly increased in the Paleolithic group (*p* < 0.01, *d* = 0.67); there was no difference between groups.

#### 3.3.2. Micronutrients

Mean pre and post intake of micronutrients for the Paleolithic and AGHE groups are shown in Table 4. Between groups, significantly greater β-carotene intake (*p* < 0.01) and lower iodine, sodium and calcium (*p* < 0.01) were observed in the Paleolithic group). Within the Paleolithic group, there were significant reductions in the intake of thiamin, riboflavin and iodine, and minerals calcium and sodium, with significant increases in vitamin C, β-carotene and total vitamin A (all *p* < 0.01 with medium to large ES) post intervention with a noticeable increase in vitamin E, and decrease in folate and iron (*p* < 0.05 with medium ES).

## 4. Discussion

The aim of the study was to assess the efficacy of the Paleolithic and AGHE diets on anthropometric, cardiovascular and metabolic risk factors during a four-week intervention and to assess energy and nutrient intakes on both dietary patterns. The use of the AGHE group in this study as a comparator group and a healthy female population has enabled effective comparison of our outcome variables with the current evidence based guidelines [3]. In terms of body composition, we have shown a 4.3% reduction in body weight and 3.8% reduction in waist circumference on a Paleolithic diet. Similar, but slightly larger changes to body composition have been reported by Ryberg *et al.*, [6], who found a 5.3% reduction in body weight and 7.5% decrease in waist circumference in ten post-menopausal women using an ad-libitum Paleolithic diet over a slightly longer, five-week period. Osterdahl [5] also used healthy subjects in a three-week intervention and found a 3.5% reduction in body weight and 2.0% drop in waist circumference. Our results showed loss of weight was significantly associated with the reduction in energy intake, and percentage of daily energy from protein. However, the reduction in body weight and fat mass found in this study cannot be attributed to the reduction in energy intake alone. This supports previous findings that lower carbohydrate diets are associated with greater losses of fat mass over a short term period [12]. Longer-term Paleolithic diets require further investigation, with poor adherence to the high protein intake shown over a two-year intervention [13].

Previous interventions using the Paleolithic diet have shown significant improvements in blood lipids [4,13,14,15] and markers of glucose metabolism [14,16,17], however, we noted reductions to both total and LDL cholesterol in both dietary groups. This suggests that the benefits to total and LDL cholesterol may be due to another factor present in both dietary patterns and not specifically an effect of the Paleolithic diet, as the changes were not related to weight loss or dietary treatment group. Reduction to saturated fat intake was common to both groups, which may in part explain our findings [18]. A recent review and meta-analysis has determined the Paleolithic diet results in greater short term improvements to markers of metabolic syndrome than dietary patterns based on broadly similar national guidelines [19]. We have not shown any changes between groups in terms of blood pressure, lipid levels or markers of glucose metabolism, due to the small sample size, the study was not sufficiently powered to detect changes in these biomarkers between groups. The use of healthy subjects and short duration of the study may have also limited our ability to detect changes. The potential impact of high protein intakes (>20% of energy from protein) over long term periods require further investigation, as the association with hyperinsulinemia and insulin resistance [20] remains unclear and a recent medium–term intervention suggested weight loss may be a more significant modifier of metabolic syndrome risk than type or quantity of protein intake [21].

Whilst there was no between group difference in energy intake post intervention, a significant reduction in energy intake was observed in the Paleolithic group, a finding consistent with previous ad-libitum Paleolithic diet studies [14,16,22]. Increases in plasma β-carotene and red cell folate in the Paleolithic group are indicative of increased fruit and vegetable consumption and may have had a positive effect on satiety [23]. Notably, our dietary analyses show reduced intake of dietary folate equivalents in the Paleolithic group, but increased red cell folate which illustrates a limitation of the food composition database to accurately estimate folate content, particularly for fruits and vegetables, where content may vary [24].

Changes to the macronutrient composition of the Paleolithic diet, with an observed reduction in carbohydrate, was expected with the removal of grain products. Supporters of the Paleolithic diet argue that genetic adaptation has not enabled effective ability to digest grains, however, the Australian Dietary Guidelines have noted the evidence base supporting the consumption of whole grain cereals, for the reduction of risk of cardiovascular disease, weight gain and type 2 diabetes, has strengthened since the previous edition of the guidelines in 2003 [3]. Although unexplored, the change in type of fibre consumed on a Paleolithic diet may have a significant effect on the gut microbiota which, in turn could alter long term health outcomes [25].

Both groups made beneficial reductions to absolute saturated fat intake, however, expressed as a percentage of daily energy from total fat, both exceeded the recommendation of 10% [26]. In the Paleolithic group, total fat as a percentage of daily energy was greater than the recommended levels of 20%–35% [26] as has been found in previous interventions using the Paleolithic diet [5]. The Food and Agriculture Organisation (FAO) has determined limited or suggestive associations between total fat intake and endometrial, breast and colorectal cancer [27]. In the Nurses’ Health and Health Professionals cohort, a low carbohydrate diet based on animal sources, was associated with higher all-cause mortality in both men and women (HR = 1.23), however, all-cause mortality was decreased if the low carbohydrate diet was based on plant sources (HR = 0.8) [28]. In a cohort of Swedish women, an increased incidence of cardiovascular disease was found (incidence ratio = 1.04) for each two-unit increase in the low carbohydrate score, irrespective of whether the protein was derived from plant or animal sources [29]. It would therefore be suggested that future, longer term interventions using *ad libitum* Paleolithic diets should focus on increasing consumption of fruits, vegetables and nuts and place limits on animal protein and fat for the individual to maximize potential health benefits.

The Paleolithic group, when compared to the AGHE group, had lower intakes of calcium, sodium and iodine and higher intakes vitamin E and β-carotene. The significant reductions in sodium and iodine intake must be interpreted with caution, as three-day WDR may not accurately record discretionary salt use, nor whether the salt was iodised. Reductions to calcium and increases in vitamin C intake were also reported by Osterdahl *et al.*, [5] using an ad-libitum Paleolithic diet. Longer term health impacts of the large reduction to calcium intake on a Paleolithic diet requires further research.

## 5. Conclusions

In healthy females, the Paleolithic diet induced a more favourable effect on body composition over the short term intervention period. The reduction in carbohydrate consumption did not impact on fibre intake in the Paleolithic group, however, significant reductions in thiamin, riboflavin and calcium were noted. We observed no significant differences between groups for cardiovascular and metabolic risk factors and further, larger studies are recommended to assess the impact of the diets over a longer term period.

## Figures and Tables

**Table 1:** Mean pre and post intervention anthropometric measurements, showing mean changes within and between groups ^1^.

Anthropometric Measure	Paleolithic (*n* = 22)			AGHE (*n* = 17)			
	Pre (Mean ± SD)	Post (Mean ± SD)	Change within Group (95% CI)	Pre (Mean ±SD)	Post (Mean ± SD)	Change within Group (95% CI)	Change between Groups Mean Difference (95% CI)
Body weight (kg)	73.8 ± 13.3	70.6 ± 12.7	−3.20 (−3.8, −2.6) **	73.0 ± 12.3	71.8 ± 12.3	−1.21 (−2.0, −0.5) **	−1.99 (−2.9, −1.0) **
Fat Mass (%)	34.1 ± 8.9	32.6 ± 9.1	−1.48 (−2.2, −0.8) **	31.5 ± 6.2	31.3 ± 6.3	−0.14 (−1.1, 0.9)	−1.34 (−2.5, −0.2) *
Fat Mass (kg)	26.2 ± 10.8	24.1 ± 10.4	−2.14 (−2.7, −1.6) **	23.6 ± 7.5	23.1 ± 7.7	−0.46 (−1.4, 0.4)	−1.68 (−2.7, −0.7) **
Fat Free Mass (%)	65.9 ± 8.9	67.0 ± 9.1	1.07 (0.3, 1.8) **	68.5 ± 6.2	68.6 ± 6.3	0.12 (−0.9, 1.1)	0.94 (−0.2, 2.1)
Fat Free Mass (kg)	48.5 ± 5.0	47.1 ± 4.7	−1.38 (−2.1, −0.6) **	50.2 ± 7.2	49.4 ± 6.5	−0.79 (−1.5, −0.1) *	−0.59 (−1.6, 0.5)
Percent Weight Loss (%)	-	−4.28 ± 1.7	-	-	−1.66 ± 1.9	-	−2.63 (−3.8, −1.5) **
Waist Circumference (cm) ^2^	85.9 ± 14.9	82.5 ± 14.1	−3.35 (−4.2, −2.5) **	83.0 ± 9.0	81.4 ± 8.6	−1.55 (−2.6, −0.4) *	−1.9 (−3.2, −0.8) **
Systolic BP (mmHg)	119 ± 14.5	115 ± 11.5	−3.32 (−9.1, 2.5)	115 ± 11.1	114 ± 9.9	−0.71 (−5.2, 3.8)	−2.61 (−10.1, 4.9)
Diastolic BP (mmHg)	77.8 ± 9.0	75.9 ± 10.0	−1.87 (−6.4, 2.7)	71.7 ± 6.7	73.4 ± 7.2	1.65 (−1.0, 4.3)	−3.51 (−9.0, 2.0)

* *p*-Value < 0.05; ** *p*-Value < 0.01; ^1^ Results calculated using paired *t*-tests within groups and Independent *t*-tests between groups; ^2^ Non-normally distributed data calculated using Mann-Whitney U (MWU) test, result shows median ± IQR. Note that the 95% CI corresponding to the MWU test is given for the median difference and was determined using the Hodges-Lehmann estimator in SPSS. AGHE: the Australian Guide to Healthy Eating.

**Table 2:** Mean pre and post biochemistry, showing mean changes within and between groups ^1^.

Biochemical Measure	Paleolithic (*n* = 22)			AGHE (*n* = 17)			Mean Difference between Groups (95% CI)
	Pre (Mean ± SD)	Post (Mean ± SD)	Change within Group (95% CI)	Pre (Mean ± SD)	Post (Mean ± SD)	Change within Group (95% CI)	
Total Plasma Cholesterol (mmol/L)	5.59 ± 1.2	5.05 ± 1.0	−0.55 (−0.8, −0.3) **	5.28 ± 1.0	4.86 ± 0.8	−0.41 (−0.7, −0.1) **	−0.13 (−0.5, 0.2)
Triglycerides (TG) (mmol/L)	0.97 ± 0.5	0.79 ± 0.3	−0.18 (−0.3, 0.0) **	0.79 ± 0.2	0.73 ± 0.2	−0.07 (−0.2, 0.0)	−0.14 (−0.4, 0.2)
Low density lipoprotein (LDL) Cholesterol ^1^ (mmol/L)	3.50 ± 1.2	3.10 ± 0.9	−0.40 (−0.6, −0.2) **	3.19 ± 0.9	2.93 ± 0.7	−0.26 (−0.5, 0.0) *	−0.11 (−0.3, 0.1)
High density lipoprotein (HDL) Cholesterol (mmol/L)	1.66 ± 0.3	1.58 ± 0.3	−0.08 (−0.2, 0.0)	1.72 ± 0.3	1.59 ± 0.3	−0.12 (−0.2, −0.1) **	0.04 (−0.1, 0.1)
Chol/HDL Ratio	3.56 ± 1.4	3.33 ± 1.0	−0.23 (−0.5, 0.0)	3.13 ± 0.7	3.15 ± 0.7	0.01 (−0.1, 0.2)	−0.24 (−0.5, 0.1)
Plasma C-Reactive Protein (mg/L)	2.23 ± 2.9	1.64 ± 1.7	−0.51 (−1.1, 0.1)	1.42 ± 1.1	1.49 ± 1.7	0.06 (−0.9, 1.0)	−0.57 (−1.6, 0.5)
Fasting Plasma Glucose (mmol/L)	4.77 ± 0.8	4.70 ± 0.6	−0.06 (−0.3, 0.1)	4.55 ± 0.4	4.49 ± 0.6	−0.05 (−0.3, 0.2)	−0.01 (−0.3, 0.3)
Fasting Serum Insulin (mU/L)	4.36 ± 2.5	4.32 ± 2.4	−0.04 (−1.2, 1.1)	4.65 ± 1.8	4.88 ± 1.9	0.23 (−0.8, 1.2)	−0.28 (−1.8, 1.3)
Serum α-carotene (μmol/L)	0.39 ± 0.4	0.46 ± 0.2	0.08 (0.0, 0.2)	0.52 ± 0.4	0.48 ± 0.2	−0.05 (−0.2, 0.1)	0.12 (−0.1, 0.3)
Serum β-carotene (μmol/L)	1.54 ± 1.3	2.41 ± 1.8	0.95 (−0.2, 1.7) *	1.53 ± 0.8	1.62 ± 1.1	0.09 (−0.3, 0.4)	0.86 (0.0, 1.7) *
Serum Lycopene (μmol/L)	0.61 ± 0.3	0.52 ± 0.2	−0.07 (−0.2, 0.0)	0.58 ± 0.2	0.50 ± 0.2	0.05 (−0.2, 0.3)	−0.12 (−0.4, 0.1)
Red Cell Folate (nmol/L)	894 ± 214	981 ± 300	86.7 (23.9, 149.5) **	758 ± 287	751 ± 218	−6.88 (−71.1, 57.3)	93.54 (7.0, 180.1) *
Plasma Creatinine (μmol/L)	70.86 ± 8.5	73.48 ± 10.8	2.52 (−0.4, 5.5)	68.4 ± 8.2	67.77 ± 8.7	−0.65 (−4.0, 2.8)	3.17 (−1.1, 7.5)
24 h Urine Sodium (mmol/day)	95.34 ± 43.1	79.3 ± 50.7	−16.1 (−36.7, 4.6)	94.8 ± 45.5	99.5 ± 44.4	4.69 (−20.0, 29.4)	−25.35 (−57.2, 6.5)
24 h Urine Creatinine (mmol/day)	10.8 ± 4.8	11.9 ± 4.6	1.04 (−1.2, 3.3)	10.2 ± 3.6	10.5 ± 2.8	0.34 (−1.0, 1.7)	0.27 (−2.5, 3.1)

* p-Value < 0.05; ** *p*-Value < 0.01; ^1^ Results calculated using paired *t*-test within groups and independent *t*-tests between groups; ^2^ LDL cholesterol reported is calculated LDL.

**Table 3:** Mean pre and post macronutrient intake, showing mean changes within and between groups ^1^.

Macronutrient	Paleolithic (*n* = 22)			AGHE (*n* = 17)			Mean Difference between Groups (95% CI)
	Pre (Mean ± SD)	Post (Mean ± SD)	Change within Group (95% CI)	Pre (Mean ± SD)	Post (Mean ± SD)	Change within Group (95% CI)	
Total Energy Intake (kJ)	7798 ± 1928	5915 ± 1452	−1883 (−2772, −993) **	8088 ± 2071	6657 ± 1725	−1432 (2973, 109)	−450.94 (−2076, 1174)
Protein (g)	90.2 ± 11.1	91.2 ± 31.5	1.78 (−11.2, 14.8)	94.9 ± 24.9	83.5 ± 28.4	−11.4 (−30.4, 7.5)	13.21 (−8.3, 34.7)
Protein (% of Energy)	20.6 ± 4.9	26.8 ± 7.2	6.19 (3.4, 9.0) **	20.1 ± 3.61	21.7 ± 5.6	1.52 (−1.3, 4.3)	4.67 (0.8, 8.6) *
Carbohydrate (g)	191 ± 68.0	103 ± 32.4	−87.8 (−115.4, −60.2) **	188 ± 62.1	163 ± 46.5	−24.9 (−58.6, 8.8)	−62.88 (−104.6, −21.2) **
Carbohydrate (% of Energy)	39.1 ± 6.2	27.8 ± 8.05	−11.3 (−15.0, −7.6) **	37.2 ± 7.67	40.6 ± 9.4	3.34 (0.1, 6.5) *	−14.63 (−19.5, −9.7) **
Total Fat (g)	71.3 ± 22.8	64.7 ± 26.7	−6.62 (−18.4, 5.2)	78.7 ± 24.1	60.1 ± 24.1	−18.5 (−38.4, 1.0)	11.92 (−9.0, 32.9)
Total Fat (% of Energy)	33.6 ± 5.3	39.8 ± 9.6	6.20 (2.0, 10.4) **	35.8 ± 5.5	32.6 ± 7.3	−3.20 (−7.1, 0.7)	9.39 (3.7, 15.1) **
Total Sugars (g)	92.5 ± 42.0	77.2 ± 26.1	−15.2 (−32.4, 1.9)	88.8 ± 29.2	75.7 ± 32.5	−13.1 (−31.1, 4.9)	−2.18 (−26.5, 22.1)
Saturated Fat (g)	26.7 ± 10.4	18.8 ± 7.3	−7.86 (−12.1, 3.7) **	31.4 ± 12.0	22.1 ± 9.2	−9.31 (−17.4, 1.2) *	1.44 (−6.8, 9.7)
Saturated Fat (% of Energy)	12.4 ± 2.5	11.9 ± 3.8	−0.52 (−2.2, 1.1)	14.1 ± 3.0	12.0 ± 2.8	−2.10 (−3.9, −0.3)	1.58 (−0.8, 4.0)
Monounsaturated Fat (g)	26.3 ± 7.9	29.5 ± 16.8	3.18 (−3.7, 10.1)	28.4 ± 9.9	22.3 ± 9.9	−6.11 (−14.2, 2.0)	9.28 (−1.0.19.6)
Polyunsaturated Fat (g)	12.8 ± 6.0	10.8 ± 6.4	−1.97 (−4.0, 0.1)	12.5 ± 4.9	10.4 ± 5.8	−2.13 (−5.7, 1.4)	0.16 (−3.6, 3.9)
Dietary Fibre (g)	24.6 ± 6.1	24.6 ± 7.2	0.05 (−3.7, 3.8)	25.7 ± 6.2	24.9 ± 10.6	−0.82 (−6.4, 4.8)	0.87 (−5.4, 7.1)
Fibre Density (%) (Fibre (g)/Energy kJ × 100)	0.33 ± 0.1	0.42 ± 0.1	0.10 (0.0, 0.2) **	0.33 ± 0.1	0.37 ± 0.1	0.04 (0.0, 0.1)	0.06 (0.0, 0.1)
Alcohol (g)	8.39 ± 8.8	1.78 ± 3.4	−6.60 (−10.5, −2.7) **	7.11 ± 8.1	3.05 ± 6.7	−4.06 (−7.1, −1.0) *	−2.54 (−7.5, 2.5)

* *p*-Value < 0.05; ** *p*-Value < 0.01; ^1^ Results calculated using paired *t*-tests within groups and Independent *t*-tests between groups.

**Table 4:** Mean pre and post intervention daily micronutrient intake, showing mean changes within and between groups ^1^.

Micronutrient	Paleolithic (*n* = 22)			AGHE (*n* = 17)			Mean Difference between Groups (95% CI)
	Pre (Mean ± SD)	Post (Mean ± SD)	Change within Group (95% CI)	Pre (Mean ± SD)	Post (Mean ± SD)	Change within Group (95% CI)	
Thiamin (mg)	1.55 ± 0.6	0.96 ± 0.5	−0.59 (−0.9, −0.3) **	1.46 ± 0.6	1.49 ± 1.1	0.04 (−0.6, 0.6)	−0.63 (−1.2, −0.01) *
Riboflavin (mg)	1.91 ± 0.6	1.47 ± 0.4	−0.44 (−0.7, −0.1) **	2.12 ± 0.7	2.11 ± 1.2	−0.01 (−0.6, 0.6)	−0.43 (−1.0, 0.8)
Niacin Equivalents (mg)	42.57 ± 6.6	43.76 ± 19.4	1.19 (−6.6, 9.0)	47.9 ± 16	44.4 ± 17	−3.52 (−13.7, 6.7)	4.71 (−7.5, 16.9)
Vitamin C (mg)	107.41 ± 50	167.72 ± 84.2	60.31 (19.6, 101.0) **	104.1 ± 50	95.6 ± 51	−8.51 (−43.0, 25.9)	68.82 (15.2, 122.5) *
Vitamin E (mg)	9.81 ± 2.5	14.25 ± 7.3	4.44 (0.9, 8.0) *	11.04 ± 3.8	9.00 ± 5.0	−2.05 (−5.2, 1.1)	6.49 (1.8, 11.2) **
Folate Equivalents (μg)	396 ± 136	316.59 ± 84.9	−78.99 (−143.7, −14.3) *	376 ± 144	350 ± 132	−25.75 (−131.0, 79.5)	−53.24 (−167.0, 60.5)
β-Carotene Equivalents (μg)	4257.94 ± 2507.5	11,379.27 ± 7966.3	7121.33 (3325.3, 10917.3) **	4042 ± 3006	4386 ± 3642	344.40 (−1940.5, 2629.3)	6776.93 (2143.7, 11410.2) **
Total Vit A Equivalents (μg)	1014.78 ± 448.3	2031.93 ± 1359.0	1017.15 (376.6, 1657.7) **	968 ± 525	984 ± 679	15.79 (−402.9, 434.5)	1001.36 (207.7, 1795.0) *
Iodine (μg)	120.04 ± 38.9	63.91 ± 22.7	−56.12 (−77.7, −34.5) **	123 ± 44	145 ± 40	−8.26 (−32.2, 15.7)	−47.86 (−79.2, −16.5) **
Sodium (mg)	2173.63 ± 568.3	915.07 ± 488.8	−1258.55 (−1553.5, −963.6) **	2082 ± 804	1878 ± 659	−203.47 (−712.4, 305.4)	−1055.09 (−1592.6, −517.6) **
Magnesium (mg)	359.14 ± 85.1	327.88 ± 96.7	−31.27 (−82.3, 19.8)	399 ± 98	366 ± 106	−32.59 (−100.7, 35.5)	1.32 (−79.1, 81.8)
Potassium (mg)	3124.18 ± 691.9	3371.21 ± 891.2	247.03 (−187.7, 681.8)	3389 ± 717	2947 ± 863	−442.66 (−1034.2, 148.9)	689.69 (−2.1, 1381.5)
Calcium (mg)	771.21 ± 204.3	355.42 ± 91.2	−415.78 (−517.4, −314.2) **	883 ± 300	759 ± 247	−123.41 (−311.0, 64.1)	−292.38 (−485.8, −99.0) **
Iron (mg)	12.69 ± 2.6	11.08 ± 2.7	−1.61 (−3.0, −0.2) *	11.8 ± 2.9	10.56 ± 3.2	−1.27 (−3.4, 0.8)	−0.34 (−2.7, 2.0)
Zinc (mg)	11.45 ± 2.4	11.86 ± 3.4	0.42 (−0.9, 1.8)	12.5 ± 4.4	10.3 ± 3.1	−2.21 (−5.0, 0.6)	2.63 (−0.1, 5.4)

* *p*-Value < 0.05; ** *p*-Value < 0.01; ^1^ Results calculated using paired *t*-tests within groups and Independent *t*-tests between groups.4. Discussion.

## Abbreviations

The following abbreviations are used in this manuscript:

AGHE	Australian Guide to Healthy Eating
PDE	Percentage of Dietary Energy
WDR	Weighed Diet Record
ES	Effect Size
